# Predicting the in vivo developmental toxicity of benzo[a]pyrene (BaP) in rats by an in vitro–in silico approach

**DOI:** 10.1007/s00204-021-03128-7

**Published:** 2021-08-25

**Authors:** Danlei Wang, Maartje H. Rietdijk, Lenny Kamelia, Peter J. Boogaard, Ivonne M. C. M. Rietjens

**Affiliations:** 1 Division of Toxicology, Wageningen University and Research, Stippeneng 4, 6708 WE Wageningen, The Netherlands; 2 Shell Health, Shell International B.V., Carel van Bylandtlaan 16, 2596 HR The Hague, The Netherlands

**Keywords:** Physiologically based kinetic (PBK) model, Alternatives for animal testing, Quantitative in vitro–in vivo extrapolation (QIVIVE), Benzo[a]pyrene (BaP)

## Abstract

Developmental toxicity testing is an animal-intensive endpoints in toxicity testing and calls for animal-free alternatives. Previous studies showed the applicability of an in vitro–in silico approach for predicting developmental toxicity of a range of compounds, based on data from the mouse embryonic stem cell test (EST) combined with physiologically based kinetic (PBK) modelling facilitated reverse dosimetry. In the current study, the use of this approach for predicting developmental toxicity of polycyclic aromatic hydrocarbons (PAHs) was evaluated, using benzo[a]pyrene (BaP) as a model compound. A rat PBK model of BaP was developed to simulate the kinetics of its main metabolite 3-hydroxybenzo[a]pyrene (3-OHBaP), shown previously to be responsible for the developmental toxicity of BaP. Comparison to in vivo kinetic data showed that the model adequately predicted BaP and 3-OHBaP blood concentrations in the rat. Using this PBK model and reverse dosimetry, a concentration–response curve for 3-OHBaP obtained in the EST was translated into an in vivo dose–response curve for developmental toxicity of BaP in rats upon single or repeated dose exposure. The predicted half maximal effect doses (ED_50_) amounted to 67 and 45 mg/kg bw being comparable to the ED_50_ derived from the in vivo dose–response data reported for BaP in the literature, of 29 mg/kg bw. The present study provides a proof of principle of applying this in vitro–in silico approach for evaluating developmental toxicity of BaP and may provide a promising strategy for predicting the developmental toxicity of related PAHs, without the need for extensive animal testing.

**Supplementary Information:**

The online version contains supplementary material available at 10.1007/s00204-021-03128-7.

## Introduction

The Registration, Evaluation, Authorization and Restriction of Chemicals (REACH) legislation requires all chemical substances produced or sold within the European Union (EU) at a volume of ≥ 100 tonnes/year to be evaluated for developmental toxicity. Developmental toxicity testing is one of the most animal-intensive endpoints in toxicity testing, estimated to require more than 20% of all animals used for toxicity testing under REACH (Jagt et al. 2004). REACH acknowledges the need for alternative, animal-free test methods, contributing to the 3Rs (replacement, reduction, refinement) of use of experimental animals in toxicological risk assessment.

Three in vitro test methods are currently scientifically validated for developmental toxicity testing: the limb bud micro mass (MM), the whole embryo culture (WEC) and the mouse embryonic stem cell test (EST) (Genschow et al. 2002, 2004). Only the EST is considered animal-free, as it makes use of the mouse embryonic stem cell line D3 (ES-D3) (Buesen et al. 2009). The differentiation assay of the EST evaluates the effect of a compound on the differentiation of ES-D3 cells into beating cardiomyocytes.

However, use of in vitro assays like the EST generates in vitro concentration–response curves, while for toxicological risk assessment, dose–response curves are needed since they enable definition of so-called points of departure (PoDs) to define health-based guidance values for safe human exposure. In vitro concentration–response curves can be translated into in vivo dose–response curves using physiologically based kinetic (PBK) modelling-based reverse dosimetry. This approach was previously shown to adequately predict in vivo developmental toxicity of various compounds, using concentration–response data from the EST (Li et al. 2017a; Louisse et al. 2010; Strikwold et al. 2017). The validity of this in vitro–in silico method for polycyclic aromatic hydrocarbons (PAHs) was not yet investigated. This although PAH-containing substances make up a large group of compounds for which REACH legislation dictates developmental toxicity testing. The aim of the present study was to evaluate the use of this in vitro–in silico approach to predict the developmental toxicity of benzo(a)pyrene (BaP). BaP was chosen as the model compound for PAHs, because BaP is well studied, and assumed to induce developmental toxicity in rats (Archibong et al. 2002; Bui et al. 1986; Feuston et al. 1989, 1994, 1996; Feuston and Mackerer 1996; Hood et al. 2000; Wu et al. 2003). Furthermore, in vivo kinetic rat data are available in the literature for BaP and its metabolite 3-hydroxybenzo[a]pyrene (3-OHBaP) (Marie et al. 2010; Moreau and Bouchard 2015) as well as in vivo dose–response data for reproductive toxicity of BaP in rats (Archibong et al. 2002; Bui et al. 1986), enabling evaluation of the predictions made by the developed in vitro–in silico approach.

BaP is well known for its bioactivation to diol epoxide metabolites that lead to DNA damage-induced carcinogenicity. For induction of developmental toxicity, BaP needs bioactivation to 3-OHBaP as shown in previous in vitro EST studies (Kamelia et al. 2020). To facilitate the prediction of tissue concentrations for 3-OHBaP, a sub-model for this metabolite was included in the PBK model. Previously, PBK models for BaP and 3-OHBaP have been developed (Campbell et al. 2016; Crowell et al. 2011; Heredia-Ortiz and Bouchard 2013; Heredia-Ortiz et al. 2011; Heredia Ortiz et al. 2014). However, these models were not applied for reverse dosimetry, leaving the question whether PBK modelling-based reverse dosimetry is suited to predict in vivo developmental toxicity of BaP.

To answer this question, in the present study a PBK model of BaP in rat was developed for predicting blood concentrations of 3-OHBaP. The model was used to translate concentration–response data for 3-OHBaP from the EST to predict an in vivo dose–response curve for developmental toxicity of BaP in rats and results obtained were compared to available data in the literature on kinetics and developmental toxicity of BaP and 3-OHBaP.

## Materials and methods

### Materials

3-OHBaP was ordered from Toronto Research Chemicals (TRC) Canada (North York, Canada). 3’-phosphate 5’-phosphosulfate (PAPS) lithium salt was purchased from Santa Cruz Biotechnology (Dallas, Texas, United States), BaP, nicotinamide adenine dinucleotide phosphate (NADPH), sodium salt, sodium phosphate, sodium chloride and Trizma^®^ base (TRIS) were purchased at Sigma-Aldrich (Zwijndrecht, The Netherlands). Uridine 5’-diphosphoglucuronic acid (UDPGA) trisodium salt was purchased from Carbosynth (Compton, United Kingdom). Pooled liver and lung S9 fractions and microsomes from male Sprague–Dawley (SD) rats were ordered from Tebu-Bio (Heerhugowaard, The Netherlands). Acetonitrile (ACN) was purchased from Biosolve (Dieuze, France). Dimethyl sulfoxide (DMSO) was obtained from Acros Organics (Geel, Belgium). Potassium hydrogen phosphate (K_2_HPO_4_) and trifluoroacetic acid (TFA) were purchased from Merck (Darmstadt, Germany).

### Methods

The PBK modelling-based reverse dosimetry approach consisted of the following steps: (1) defining a PBK model describing the kinetics of 3-OHBaP, the main metabolite of BaP, in rats, (2) determining kinetic parameter values for metabolism of BaP and conjugation of 3-OHBaP, (3) evaluation of the PBK model using in vivo kinetic literature data, (4) translation of in vitro concentration–response data for 3-OHBaP in the EST (Kamelia et al. 2020) into in vivo dose–response data for developmental toxicity of BaP using PBK model-facilitated reverse dosimetry, (5) evaluation of the predicted dose–response curve by comparison to the literature reported dose–response data (Archibong et al. 2002; Bui et al. 1986).

#### Development of a PBK model for BaP and 3-OHBaP in rats

**Fig. 1:**
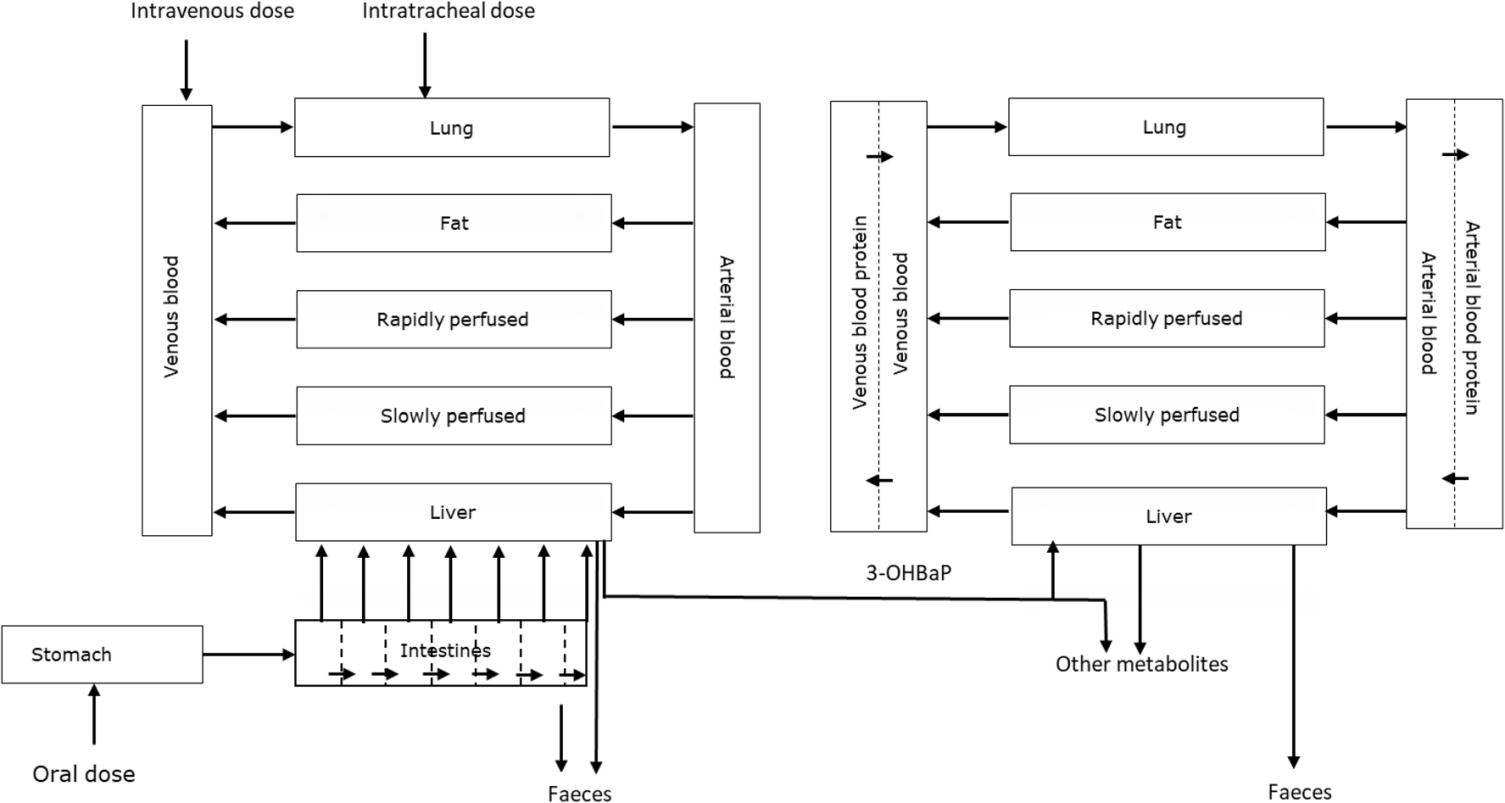
Schematic overview of the PBK model of BaP containing a sub-model for 3-OHBaP in rat (color figure online)

The PBK model was defined based on the conceptual model for BaP with a sub-model for 3-OHBaP taking into account the model codes for BaP PBK models that included sub-models for 3-OHBaP reported in the literature (Campbell et al. 2016; Crowell et al. 2011; Heredia-Ortiz and Bouchard 2013) and is presented in Fig. 1. The conceptual PBK model for BaP consisted of separate compartments for venous blood, arterial blood, fat tissue, liver tissue, lung tissue, rapidly and slowly perfused tissue, stomach and intestines. 3-OHBaP is highly lipophilic. To prevent 3-OHBaP from partitioning into the fat tissue in the model simulations, it was essential to include a blood protein compartment in the model to allow binding of 3-OHBaP to blood protein. The unbound fraction of 3-OHBaP in the blood compartment was represented by the *f*_ub, in vivo_, calculated as described in “Translating in vitro concentration–response data into in vivo dose–response data using PBK modelling-based reverse dosimetry” section. The fraction of 3-OHBaP bound to protein in the blood protein compartment (*f*_b, in vivo_) was calculated as 1 minus the *f*_ub, in vivo_. The intestinal compartment was divided into seven sub-compartments to describe the passage of BaP trough the intestines upon oral exposure (Zhang et al. 2018). Conversion of BaP into 3-OHBaP and other metabolites was initially assumed to occur in the liver and lung (Heredia-Ortiz et al. 2011). Clearance of 3-OHBaP was assumed to result from hepatic and pulmonary conjugation (Cohen 1990; Cohen and Moore 1976). The relative contribution of pulmonary metabolism of BaP and 3-OHBaP compared to hepatic metabolism of these compounds was investigated as well. Pulmonary metabolism of BaP and 3-OHBaP quantified based on incubations of BaP with rat lung microsomes and 3-OHBaP with rat lung S9, calculated as described in  "Determining kinetic parameter values for metabolism of BaP and conjugation of 3-OHBaP" section, was shown to be negligible as compared to metabolism in the liver (see "Development of a PBK model describing the kinetics of BaP and 3-OHBaP in rats" section) and thus not included in the PBK model. In the model, it is assumed that both BaP and 3-OHBaP are eliminated to the faeces by biliary excretion.

BaP needs bioactivation to 3-OHBaP to induce developmental toxicity in vitro (Kamelia et al. 2020). Therefore, the PBK model contained a sub-compartment describing the kinetics of 3-OHBaP, enabling prediction of blood concentrations of 3-OHBaP as a function of the dose of BaP, required for the reverse dosimetry. Based on the conceptual model the differential equations were defined and inserted in Berkley Madonna 8.3.18 (UC Berkeley, California, USA) using the Rosenbrock’s algorithm for stiff systems. Model equations are included in supplementary materials 1.

The 3-OHBaP sub-model consisted of the same compartments as the BaP model, except for stomach and intestine, which were not relevant for the kinetics of 3-OHBaP, since 3-OHBaP is formed in the liver. Studies in pregnant rats have shown that the blood concentration of BaP in maternal and foetal blood are similar (Withey et al. 1993). The same was found for the concentration of BaP metabolites in maternal and foetal blood (Withey et al. 1993). It was, therefore, assumed that BaP and its metabolites readily cross the placenta and that the reproductive toxicity observed in vivo is dependent on the maternal blood concentration of 3-OHBaP. For this reason, separate compartments for placental and foetal tissue were not included in the model.

Physiological and anatomical parameter values were taken from literature (Brown et al. 1997; Crowell et al. 2011) and are presented in Table 1.

**Table 1:** Physiological, anatomical and physicochemical parameter values for BaP and 3-OHBaP for the rat PBK model

Model parameter	Symbol	Value	References
Physiological parameters			
Body weight	BW	0.245^a^	Marie et al. (2010) Moreau and Bouchard (2015)
Fractional tissue volumes			
Fat	VFc	0.065	Crowell et al (2011), based on Brown et al. (1997)
Liver	VLc	0.037	
Lung	VLuc	0.005	
Arterial blood	VABc	0.0257	
Venous blood	VVBc	0.0514	
Rapidly perfused tissue	VRc	0.2159	
Slowly perfused tissue	VSc	0.6	
Cardiac output (mL/s)	QC	15*BW^0.74^	
Fractional tissue blood flows			
Fat	QFc	0.07	
Liver	QLc	0.183	
Lung	QLuc	1	
Rapidly perfused tissue	QRc	0.4	
Slowly perfused tissue	QSc	0.347	
Physicochemical parameters			
* Benzo[a]pyrene*			
Molecular weight	MWBaP	252.31	
log*P*		6.0	
Fraction unbound	*f*_ub_	0.006	Calculated according to Lobell and Sivarajah (2003)
Tissue:blood partition coefficients			
Fat	PFBAP	496.38	Crowell et al. (2011) based on Poulin and Theil (2000)
Liver	PLBAP	13.31	
Lung	PLuBAP	13.31	
Rapidly perfused tissue	PRBAP	13.31	
Slowly perfused tissue	PSBAP	6.99	
* 3-hydroxybenzo[a]pyrene*			
Molecular weight	MW3OHBaP	268.3	
log*P*		5.9	
Fraction unbound	*f*_ub_	0.007	Calculated according to Lobell and Sivarajah (2003)
Tissue:blood partition coefficients			
Fat	PF3OHBAP	401	Crowell et al. (2011) based on Poulin and Krishnan (1995)
Liver	PL3OHBAP	12.24	
Lung	PLu3OHBAP	12.24	
Rapidly perfused tissue	PR3OHBAP	12.24	
Slowly perfused tissue	P3OH BAP	6.43	

^a^Median of body weights of rats in these two studies

The tissue:blood partition coefficients for BaP and 3-OHBaP were previously calculated by Crowell et al. (2011), according to the method of Poulin and Krishnan (1995) and Poulin and Theil (2000) and were applied in the current PBK model (Table 1).

Given the nature of the in vivo data available for model evaluation, and evaluation of the predicted toxicity, the model included single and repeated intravenous, intratracheal and oral exposure to BaP. For oral exposure, stomach emptying and intestinal transfer of the parent compound were included. The uptake of BaP from the intestines to the liver was described for the seven sub-compartments using the apparent in vivo permeability coefficient (*P*_app, in vivo_) value. The *P*_app, in vivo_ was derived from the in vitro *P*_app, Caco-2_ value for BaP that was previously determined in the Caco-2 model (Goth-Goldstein et al. 1999). The *P*_app, in vivo_ was calculated using the following equation: log (*P*_app, in vivo_) = 0.6836 × log (*P*_app, Caco−2_) − 0.5579 (Sun et al. 2002) and applied in the model as described before (Zhang et al. 2018).

Hepatic conversion of BaP into 3-OHBaP and other metabolites was described by the *V*_max_ and *K*_m_, determined in vitro using incubations with rat liver microsomes. Pulmonary metabolism of BaP was shown to be not relevant for the PBK model based on results from incubations with rat lung microsomes and BaP as described in "Determining kinetic parameter values for metabolism of BaP and conjugation of 3-OHBaP" section.

Clearance of 3-OHBaP was assumed to be the result of conjugation in liver tissue. Sulfation and glucuronidation are the main contributors to 3-OHBaP clearance (Cohen 1990; Cohen and Moore 1976). Based on the results of incubation experiments described in "Determining kinetic parameter values for metabolism of BaP and conjugation of 3-OHBaP" section, pulmonary conjugation was considered irrelevant for clearance of 3-OHBaP in the PBK model. *V*_max_ and *K*_m_ values for glucuronidation and sulfation of 3-OHBaP in rat liver were determined in in vitro incubations with rat liver S9, performed as described in "Determining kinetic parameter values for metabolism of BaP and conjugation of 3-OHBaP" section.

All in vitro *V*_max_ values were scaled to microsomal or S9 protein content of rat liver using the following scaling factors: 45 mg microsomal protein per gram liver tissue and 125 mg S9 protein per gram liver tissue (Houston and Galetin 2008). The scaled *V*_max_ values were subsequently converted to *V*_max_ in nmol/min/liver using the liver weight of 9.1 g, calculated from the body weight and fractional liver weight presented in Table 1. The *K*_m_ in vitro was assumed equal to the *K*_m_ in vivo.

#### Determining kinetic parameter values for metabolism of BaP and conjugation of 3-OHBaP

##### Metabolism of BaP

The formation of 3-OHBaP and other metabolites from BaP in liver and lung tissue was investigated in incubations with rat liver microsomes and rat lung microsomes. The incubation mixtures consisted of (final concentrations) 0.1 mM potassium phosphate (pH 7.4), 5 mM MgCl_2_, 0.5 mg/ml rat liver microsomes or 2 mg/ml rat lung microsomes, and 1 mM NADPH in conical glass vials. Incubation mixtures were pre-incubated for 1 min, after which the reaction was initiated by the addition of BaP from 100 times concentrated stock solutions in DMSO to reach the final volume of 200 µl (1% DMSO v/v) with BaP concentrations ranging from 0 to 200 µM. The mixtures were incubated in a shaking water bath at 37 °C for 30 min. 20 µl ice-cold 10% (v/v) perchloric acid (HClO_4_) was added to terminate the reactions and the mixtures were put on ice for at least 15 min. Di-isopropyl ether (DIPE) was used to extract BaP and its metabolites from the incubation mixture. To this end, 1 ml DIPE was added to each incubation mixture, the tubes vortexed for 20 s and the upper layer was collected. Extraction was performed three times. Remaining DIPE was removed by evaporation under a stream of N_2_. Subsequently, extracts were re-dissolved in 100 µl methanol and transferred to UPLC vials for analysis.

##### Conjugation of 3-OHBaP

Incubations with rat liver and lung S9 fractions were optimized to establish linearity over time and protein concentrations for the rate of glucuronidation and sulfation of 3-OHBaP. The experiments performed for time optimization revealed that the pulmonary formation rate of sulfated and glucuronidated metabolites of 3-OHBaP was negligible compared to the formation rate of sulfonated and glucuronidated metabolites by the liver (see "Evaluation of the PBK model and sensitivity analysis" section). Pulmonary conjugation was, therefore, considered not relevant for in vivo clearance of 3-OHBaP in the current PBK model and no further experiment for in vitro kinetics with lung fractions were performed.

Kinetics for 3-OHBaP glucuronidation, were quantified using incubations with pooled liver S9 fractions from male SD rats. Incubation mixtures in a final volume of 200 µl in conical glass vials consisted of (final concentrations) 0.1 mM Tris–HCl (pH 7.4), 5 mM MgCl_2_, 0.1 mg/ml rat liver S9, 3 mM UDPGA and 0.025 mg/ml alamethicin.

Hepatic sulfonation of 3-OHBaP was evaluated using pooled liver S9 fractions of male SD rats, in incubation mixtures with a final volume of 200 µl in conical glass vials containing (final concentrations) 0.1 mM Tris–HCl (pH 7.4), 0.1 mg/ml rat liver S9 and 0.2 mM PAPS.

All incubation mixtures were pre-incubated for 1 min, after which the reaction was initiated by addition of 3-OHBaP in final concentrations ranging from 0.01 to 50 µM (glucuronidation) or 0.01–100 µM (sulfation) added from 100 times concentrated stock solutions in DMSO to reach the final volume of 200 µl (1% DMSO v/v). The mixtures were incubated in a shaking water bath at 37 °C for 20 min (glucuronidation) or 70 min (sulfation). 100 µl of ice-cold acetonitrile was added to terminate the reactions and the mixtures were put on ice for at least 15 min. Subsequently, the tubes were centrifuged at 4 °C and 3717 g per minute for 5 min. The supernatant was collected and analysed by Ultra Performance Liquid Chromatography (UPLC). All incubations were performed in triplicate.

##### UPLC analysis

The collected supernatants were analysed using a UPLC Nexera series (Shimadzu, Kyoto, Japan) to quantify the metabolites of BaP and the conjugates of 3-OHBaP formed in incubations with rat liver microsomes and S9, respectively. The UPLC was equipped with a Photodiode Array (PDA) detector, recording wavelengths between 190 and 400 nm and a Phenomenex C18 column (Phenomenex, Torrance, California, United States). The column temperature was kept at 40 °C and the auto-sampler at 4 °C during analysis. The mobile phase consisted of Nanopure water containing 0.1% (v/v) trifluoroacetic acid (TFA) (A) and acetonitrile containing 0.1% (v/v) TFA (B) at a flow rate of 0.3 ml/min. The total run time was 23 min and 30 s, starting with 10% B for 30 s, increasing to 100% B in 15 min, maintaining this condition for 3 min before returning to the initial conditions of 10% B in 30 s. 10 µl of sample was injected per run. Under these conditions the metabolite 3-OHBaP, detected at 258 nm, eluted at 10.16 min. For the glucuronidated and sulfonated metabolite of 3-OHBaP, retention time and detection wavelength were 7.5 min, 303.3 nm and 7.4 min, 301 nm, respectively. The amounts of 3-OHBaP in the microsomal incubation sample and of glucuronidated and sulfated 3-OHBaP in the S9 incubation samples were quantified by integrating peak areas at their respective wavelengths using a calibration curve prepared with commercially available 3-OHBaP. To obtain the *V*_max_ and *K*_m_ the in vitro data for the substrate concentration-dependent rate of metabolite formation were fitted to the Michaelis–Menten equation using Graphpad Prism 9.0.1 for Windows (GraphPad Software, San Diego, California, USA).

#### PBK model evaluation and sensitivity analysis

The PBK model performance was evaluated by comparing predicted time-dependent blood concentrations of 3-OHBaP to reported in vivo time-dependent blood concentrations in rats after dosing BaP intravenously (Marie et al. 2010; Moreau and Bouchard 2015), intratracheally, and orally (Moreau and Bouchard 2015). Model development and evaluation was focussed on accurate prediction of 3-OHBaP, because the reverse dosimetry is based on the EST data for 3-OHBaP-mediated induction of in vitro developmental toxicity. Further evaluation of the PBK model was done by comparing predicted dose–response data, obtained by reverse dosimetry of the EST data of 3-OHBaP, to in vivo dose–response data of reproductive toxicity of BaP in rats (Archibong et al. 2002; Bui et al. 1986), performed as described in "Evaluation of predicted dose-dependent developmental toxicity effect" section. Given the nature of the in vivo study used for evaluation of the predicted dose–response curve, the blood concentrations of 3-OHBaP were also predicted for repeated daily exposure to BaP, until steady state of the *C*_max_ of 3-OHBaP was reached. An overview of the characteristics of the in vivo kinetic and dose–response studies used for model evaluation is presented in Tables 2 and 3.

**Table 2:** Summary of studies on in vivo kinetics of BaP and 3-OHBaP used for evaluation of the model predicted blood concentrations

Species	Weight (g)	Compound	Dosage	Dose (mg/kg bw)	Route of exposure	References
SD rat, male	260–290	BaP	Single	10	Intravenous	Marie et al. (2010)
SD rat, male	200–250	BaP	Single Single Single Single	10 10 10 10	Intravenous Intratracheal Oral Cutaneous	Moreau and Bouchard (2015)

**Table 3:** Summary of the in vivo developmental toxicity study used for evaluation of predicted developmental toxicity of BaP using the developed PBK modelling-based reverse dosimetry approach

Species	*N*	Weight (g)	Route of exposure	Dosage	Dose (µg BaP/m^3^)	Dose (mg/kg bw/day)	Endpoint(s)	References
F-344 rat, female	10	N/A	Inhalation	Repeated, 4 h/day for 10 days	25, 75 or 100	4.75, 14.25 or 19^a^	Foetal survival per litter Implantation sites per dam Pups per litter	Archibong et al. (2002)
SD rat, female	10–15	225–250	Subcutaneous	Repeated Daily for 3 or 6 days		50	Implantations per litter Number of live and dead fetuses Number of resorptions	Bui et al. (1986)

^a^Oral dose equivalent as reported by Hood et al. (2000) and Ramesh et al. (2002)

For further evaluation of the PBK model, the parameters that were most influential for the prediction of the maximum blood concentration (*C*_max_) of 3-OHBaP upon intravenous, intratracheal and oral exposure to BaP were identified by a sensitivity analysis. The sensitivity analysis was performed for intravenous, intratracheal and oral exposure. To this end, each parameter value (*P*) was increased by 10% (*P′*), while keeping the other parameter values constant and the total fraction of arterial and venous blood flow at 1, resulting in an initial (C) and modified (*C′*) value of the model prediction for the *C*_max_ of 3-OHBaP. Sensitivity coefficients (SC) were calculated using the following equation: SC = (*C*ʹ – *C*)/(*P*ʹ – *P*) × (*P*/*C*) (Evans and Andersen 2000). The sensitivity analysis was performed for a single dose of 10 mg/kg bw BaP, as this dose was applied in the kinetic in vivo studies used for evaluation of the model predicted blood concentrations of 3-OHBaP (Marie et al. 2010; Moreau and Bouchard 2015). The median body weight of the rats in these kinetic in vivo studies was 0.245 kg and was applied in the model when performing the sensitivity analysis.

#### Translating in vitro concentration–response data into in vivo dose–response data using PBK modelling-based reverse dosimetry

The in vitro concentration–response curve obtained for 3-OHBaP in the EST (Kamelia et al. 2020) was translated into a predicted in vivo dose–response curve, using PBK modelling-based reverse dosimetry. The in vivo developmental toxicity response to BaP is assumed to depend on the *C*_max_ of unbound 3-OHBaP in the maternal rat blood (*C*_ub, in vivo_). Therefore, the *C*_ub, in vivo_ was set equal to the concentration of unbound 3-OHBaP in vitro (*C*_ub, in vitro_). To correct for differences in *f*_ub_ between rat blood (*f*_ub, in vivo_) and the EST assay medium (*f*_ub, in vitro_), the following equation was used:

$$C_{{\text{in vivo}}} = \frac{{C_{{\text{in vitro}}} *f_{{\text{ub, in vitro}}} }}{{f_{{\text{ub, in vivo}}} }},$$

where *C*_in vivo_ is the total 3-OHBaP concentration in the maternal blood, *C*_in vitro_ is the total 3-OHBaP concentration used in vitro, *f*_ub, in vitro_ is the fraction unbound in the EST assay medium and *f*_ub, in vivo_ is the fraction unbound in rat blood.

##### Calculating the fraction unbound (*f*_ub_) of 3-OHBaP in assay medium

The *f*_ub, in vivo_ of 3-OHBaP was calculated from the log*P* value of 3-OHBaP based on the method described previously (Lobell and Sivarajah 2003) using the QIVIVE tool of Wageningen Food Safety Research (WFSR) (https://wfsr.shinyapps.io/wfsrqivivetools/) (Punt et al. 2021). This in silico method assumes the *f*_ub, in vivo_ in rat plasma for rat to be the same as for human plasma. Furthermore, the *f*_ub, in vitro_ values were assumed to vary linear with the protein content in the biological matrix. This assumption is supported by the linear relationship between the unbound fraction and the albumin concentration in the in vitro test system reported previously for some chlorophenols (Gulden et al. 2002). The fractions bound in vivo (*f*_b, in vivo_) were calculated as 1 minus the *f*_ub, in vivo_. The log*P* values and calculated *f*_ub, in vivo_ and *f*_b, in vivo_ are presented in Table 1.

The *f*_ub, in vivo_ and *f*_ub, in vitro_ depend on the protein content present in rat blood plasma and assay medium, respectively. The relative amount of protein present in the assay medium used in the EST [15% (Kamelia et al. 2020)], is approximately twice the protein content of rat blood plasma (7.5% (Torbert 1935)). Therefore, the *f*_ub, in vitro_ was assumed to be half of the *f*_ub, in vivo_.

##### PBK modelling-based reverse dosimetry

Reverse dosimetry was performed to calculate the dose of BaP that would give rise to the *C*_in vivo_ of 3-OHBaP obtained by setting the in vitro unbound concentrations applied in the EST equal to the unbound in vivo concentration_,_ as described in "Translating in vitro concentration–response data into in vivo dose–response data using PBK modelling-based reverse dosimetry" section. Reverse dosimetry was performed for exposure to a single oral dose of BaP and for repeated daily intravenous and oral dosing. For repeated exposure, BaP was dosed daily until steady state of the 3-OHBaP blood concentration was reached. BaP doses were calculated using a parameter plot where the maximum blood concentration (*C*_in vivo_) of 3-OHBaP was plotted against the oral dose of BaP (mg/kg bw). In vivo dose–response data used to evaluate the model predicted dose–response curve were available from literature (Archibong et al. 2002; Bui et al. 1986), the details of these studies are summarized in Table 3.

#### Evaluation of predicted dose-dependent developmental toxicity

##### Conversion of the exposure concentration of BaP in air to an oral equivalent dose per kg bw

The dose–response data predicted from the concentration–response curves derived in the EST (Kamelia et al. 2020) were compared to in vivo dose–response data for reproductive toxicity of BaP upon nasal inhalation (Archibong et al. 2002) and to the in vivo data for reproductive toxicity of BaP upon subcutaneous injection (Bui et al. 1986). The details of these studies are summarized in Table 3. Foetal survival, calculated as the fraction of live foetuses relative to the number of implantation sites reported in these studies, was taken as measure for in vivo reproductive toxicity. Archibong et al. (2002) exposed Fisher 344 (F-344) rats to 25, 75 or 100 µg BaP/m^3^ via nasal inhalation, for 4 h per day, from gestation day 11–20. Previous studies reported that the inhalation doses of 25, 75 or 100 µg BaP/m^3^ correspond to an equivalent oral dose of 4.75, 14.25 and 19 mg/kg bw, respectively (Hood et al. 2000; Ramesh et al. 2002). These oral dose equivalents in mg/kg bw were used for the comparison with model predicted dose–response data. Bui et al. (1986) exposed pregnant SD rats to 50 mg BaP/kg bw per day via subcutaneous injection from gestation day 6–8 or 6–11.

##### Calculating the ED_50_ for evaluating the predicted dose–response data

ED_50_ values were calculated for the predicted and reported dose–response data, using the non-linear regression with three parameters in Graphpad Prism version 9.0.1 for Windows (GraphPad Software, San Diego, California USA). The ED_50_ value was calculated for the fraction differentiated into beating cardiomyocytes for the predicted dose–response data. For the in vivo studies, foetus survival (number of live foetuses as fraction of total implantations) was used as response.

## Results

### Development of a PBK model describing the kinetics of BaP and 3-OHBaP in rats

#### Kinetics of BaP and 3-OHBaP in rats

**Fig. 2:**
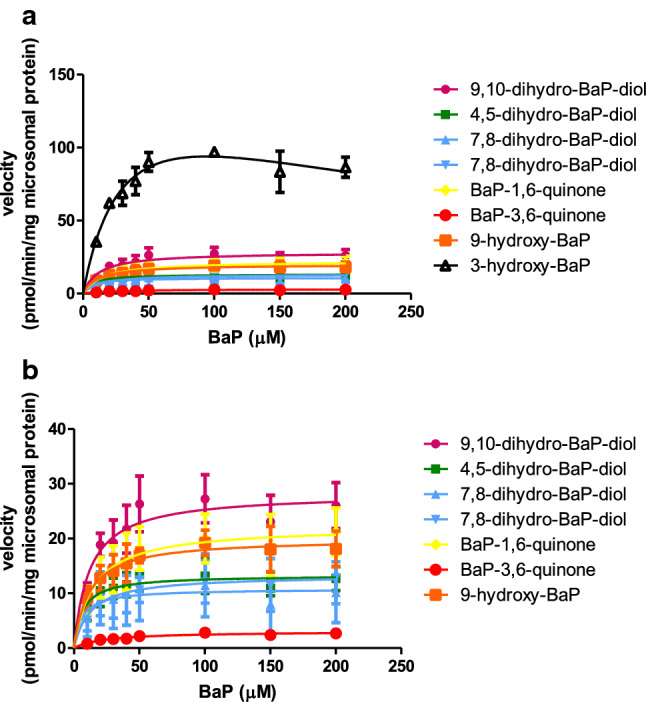
BaP concentration-dependent formation of BaP metabolites in incubations with rat liver microsomes (**a**). Each symbol represents an experimental mean and vertical bars are standard errors of the mean (*n* = 3). Triangle black line, 3-hydroxy-BaP; square orange line, 9-hydroxy-BaP; dot red line, BaP-3,6-quinone; diamond yellow line, BaP-1,6-quinone; both up and down pointing triangle blue line, 7,8-dihydro-BaP-diol (isomers); square green line, 4,5-dihydro-BaP-diol; dot pink line, 9,10-dihydro-BaP-diol. To reduce overlap of the respective curves, Fig. 1b presents the data for all metabolites except 3-hydroxyBaP on a different *y*-axis scale (color figure online)

Figure 2 presents the BaP concentration-dependent formation of metabolites in incubations with rat liver microsomes. Table 4 shows the kinetic parameter values, *V*_max_, *K*_m_ and catalytic efficiency that were derived from these data. The metabolites of B[a]P were characterized based on the elution order and reference UV spectra reported in the literature (Chou 1983; Hamernik 1984; Hamernik et al. 1983; Koehl et al. 1996; Moserova et al. 2009; Veignie et al. 2002; Yang et al. 1975) and commercially available reference chemicals. Three cis-B[a]P dihydrodiols were identified as B[a]P-9,10-dihydrodiol, B[a]P-4,5-dihydrodiol and B[a]P-7,8-dihydrodiol with identical UV spectra to those reported previously (Chou 1983; Hamernik 1984; Hamernik et al. 1983). Another B[a]P-dihydrodiol was identified as a geometric isomer being trans-B[a]P-7,8-dihydrodiol due to spectral similarity to that of cis-B[a]P-7,8-dihydrodiol. Two quinones of B[a]P were identified as BaP-1,6-quinone and B[a]P-3,6-quinone with reported identical spectra (Chou 1983; Veignie et al. 2002). 9-Hydroxy-B[a]P was identified according to the reported UV wavelengths of 9-hydroxy-B[a]P (Sims 1968). 3-OHB[a]P was identified by co-elution and identical spectra to that of commercially available 3-OHBaP and the spectra reported by Hamernik (1984).

**Table 4:** Metabolites of BaP formed in incubations with rat liver microsomes and the corresponding kinetic parameters

Metabolites	Structure	*K*_m_, µM	*V*_max_, pmol/min/mg microsomal protein	Catalytic efficiency (*V*_max_/*K*_m_), µl/min/mg protein
9,10-dihydro-B[a]P-diol		11.9 ± 5.5	28.3 ± 2.8	2.4
4,5-dihydro-B[a]P-diol		6.5 ± 4.5	13.3 ± 1.4	2.0
7,8-dihydro-B[a]P-diol		14.6 ± 9.8	7.0 ± 1.1	0.5
7,8-dihydro-B[a]P-diol		12.6 ± 10.7	13.3 ± 2.5	1.1
B[a]P-1,6-quinone		14.8 ± 8.8	22.2 ± 3.2	1.5
B[a]P-3,6-quinone		24.0 ± 14.4	3.0 ± 0.5	0.1
9-hydroxy-B[a]P		11.4 ± 5.2	20.0 ± 1.9	1.8
3-hydroxy-B[a]P		34.2 ± 17.7	162.2 ± 47.6	7.8

**Fig. 3:**
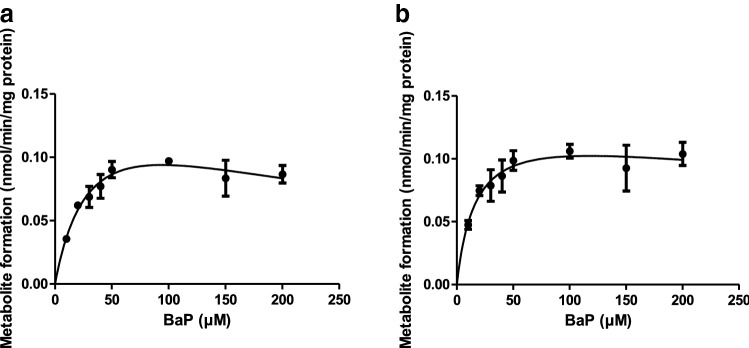
Concentration-dependent oxidation of BaP to (**a**) 3-OHBaP and (**b**) sum of other metabolites in incubation with rat liver microsomes. Symbols represent the mean of three independent experiments, the error bars represent the standard error of the mean (SEM)

Given these results, in the PBK model, the metabolism of BaP was described by two Michaelis Menten equations, one to describe the bioactivation of BaP to 3-OHBaP, and the other to describe the combined conversion to all other metabolites together. Figure 3 presents the corresponding curves and Table 5 shows the kinetic parameter values, *V*_max_ and *K*_m_, derived from these data. The *V*_max_ of 3-OHBaP formation and the *V*_max_ for the sum of formation of all other minor metabolites were 0.16 and 0.13 nmol/min/mg microsomal protein, respectively, amounting to 4.1 and 3.3 µmol/min/liver, when scaled to the whole liver using the scaling factor described in "Determining kinetic parameter values for metabolism of BaP and conjugation of 3-OHBaP" section.

**Table 5:** Kinetic parameter values for liver metabolism of BaP and 3-OHBaP in rat

	*V*_max_^a^	*K*_m_^b^	Scaled *V*_max_^c^	Scaled *V*_max_^d^
BaP to 3-OHBaP	0.16	34	0.44	4.1
BaP to other metabolites	0.13	17	0.36	3.3

	*V*_max_^e^	*K*_m_ (µM)^b^	Scaled *V*_max_^c^	Scaled *V*_max_^d^
Glucuronidated 3-OHBaP	5.7	10	43	394
Sulfated 3-OHBaP	0.48	17	3.6	33

^a^nmol/min/mg microsomal protein

^b^µM

^c^µmol/h/g liver

^d^µmol/h/liver

^e^nmol/min/mg S9 protein

**Fig. 4:**
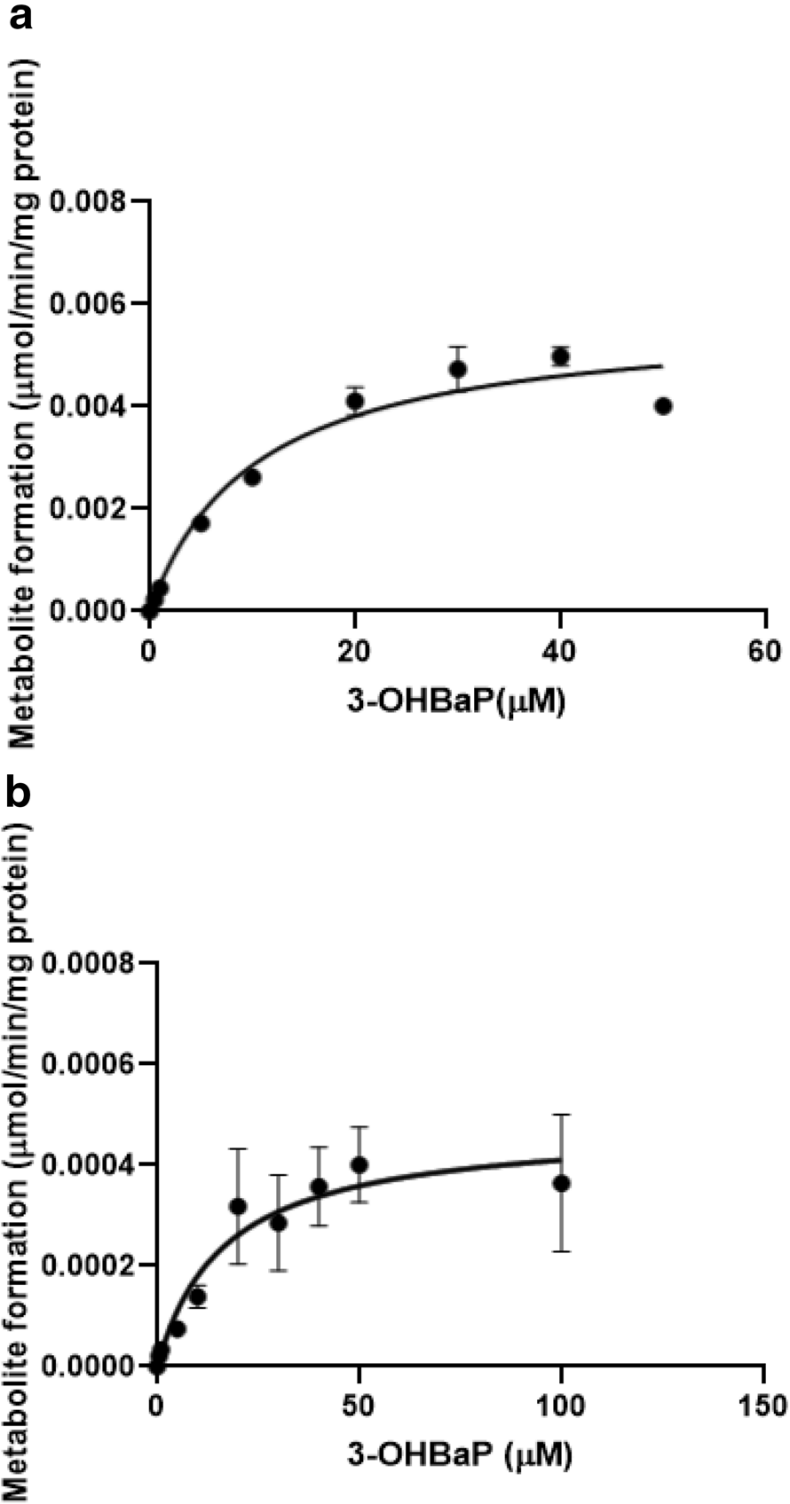
Concentration-dependent glucuronidation (**a**) and sulfation (**b**) of 3-OHBaP in incubations with rat liver S9. Symbols represent the mean of three independent experiments, the error bars represent the standard error of the mean (SEM)

Figure 4 shows the concentration-dependent rate of (a) glucuronidation and (b) sulfation of 3-OHBaP in incubations with rat liver S9. Hepatic glucuronidation and sulfation of 3-OHBaP followed Michaelis–Menten kinetics. Substrate inhibition occurred at concentrations of 50 µM and higher for glucuronidation. Table 5 shows the kinetic parameter values, *V*_max_ and *K*_m_, derived from these data. Incubations of BaP with lung microsomes did not result in formation of detectable levels of 3-OHBaP, except for the incubations with the highest concentration of BaP (200 µM). The rate of 3-OHBaP formation at 200 μM BaP in rat lung microsomes amounted to 0.81 pmol/min/mg protein converted with a scaling factor of 3.67 mg microsomal protein/g lung and a lung weight of 1.25 g (Table 1) to a rate of conversion of 3.7 pmol/min/lung amounting to 0.01% of the rate of conversion at 200 μM BaP in the liver of 86.6 pmol/min/mg microsomal protein, amounting to a rate of conversion of 36.0 × 10^3^ pmol/min/liver using the scaling factors for microsomal protein content of the liver and liver weight mentioned in "Determining kinetic parameter values for metabolism of BaP and conjugation of 3-OHBaP" section. Based on this result, it was concluded that 3-OHBaP formation from BaP in the lung does not add substantially to the overall 3-OHBaP formation and is not to be included in the PBK model.

Incubations of 3-OHBaP with rat lung S9 and 50 µM 3-OHBaP showed that the formation rate of glucuronidated metabolites was 0.015 nmol/min/mg S9 protein, scaled to the whole lung with a scaling factor of 10.19 mg S9 protein/g lung and a lung weight of 1.25 g to a conversion rate of 0.19 nmol/min/lung. For liver, the glucuronidation rate at 50 μM 3-OHBaP was 4.0 nmol/min/mg S9 protein, amounting to 4.6 × 10^3^ nmol/min/liver, scaled to the whole liver using the scaling factor described in "Determining kinetic parameter values for metabolism of BaP and conjugation of 3-OHBaP" section. This implies that at 50 µM 3-OHBaP the pulmonary conversion rate amounted to approximately 0.04% of the hepatic conversion rate. For sulfonation, the estimated conversion rate in incubations with rat lung S9 and 50 µM 3-OHBaP was 0.001 nmol/min/mg S9 protein, amounting to 0.013 nmol/min/lung using the scaling factors mentioned above. For liver, the sulfation rate of 3-OHBaP at 50 μM 3-OH-BaP was 0.40 nmol/min/mg protein, scaled to the whole liver applying the scaling factors mentioned in "Determining kinetic parameter values for metabolism of BaP and conjugation of 3-OHBaP" section, resulting in 464 nmol/min/liver. Thus, for sulfonation, the pulmonary conversion rate is < 0.003% of the hepatic conversion rate. Based on these results, it was concluded that pulmonary conjugation of 3-OHBaP was negligible and, therefore, did not need to be included in the PBK model.

### Evaluation of the PBK model and sensitivity analysis

**Fig. 5:**
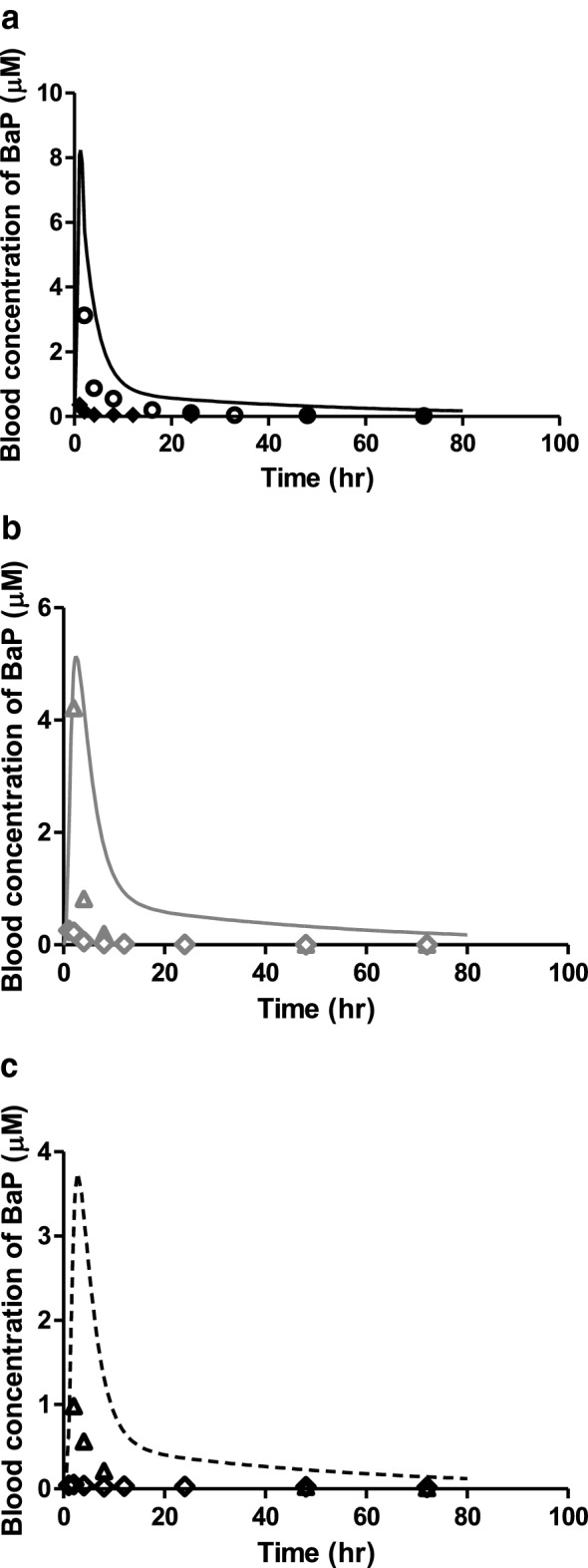
Reported and predicted blood concentrations of BaP in rats upon (**a**) intravenous, (**b**) intratracheal and (**c**) oral exposure to 10 mg/kg BaP in rats. Symbols represent (**a**) the average blood concentrations reported in the in vivo studies of Moreau and Bouchard (2015) (filled diamonds) and Marie et al. (2010) (open circles) for intravenous exposure, (**b**) the average blood concentrations reported in the in vivo study of Moreau and Bouchard (2015) (grey open diamonds) and scaled Moreau and Bouchard data based on the fold difference observed in intravenous data (grey open triangles) upon intratracheal exposure, and (**c**) the average blood concentrations reported in the vivo study of Moreau and Bouchard (2015) (black open diamonds) and scaled Moreau and Bouchard data based on the fold difference observed in intravenous data (black open triangles) upon oral exposure. The lines represent the model predicted blood concentrations upon (**a**) intravenous (black solid line), (**b**) intratrachael (grey solid line) and (**c**) oral (black dashed line) exposure

**Fig. 6:**
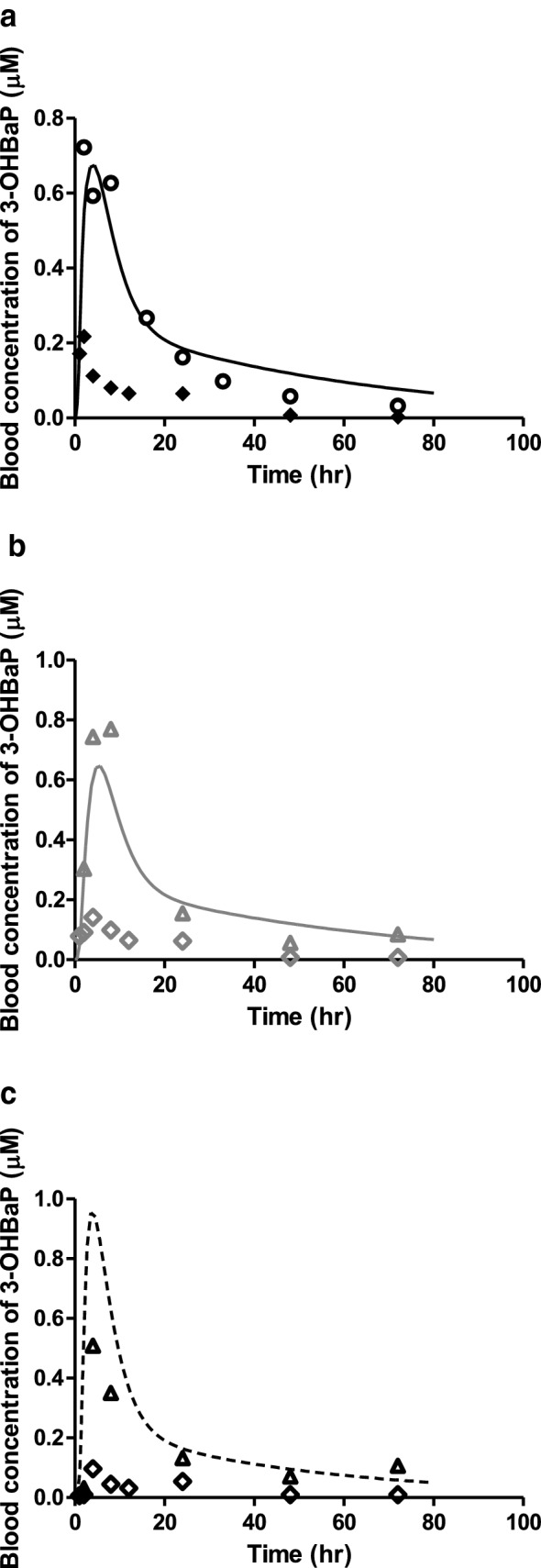
Reported and predicted blood concentrations of 3-OHBaP in rats upon (**a**) intravenous, (**b**) intratracheal and (**c**) oral exposure to 10 mg/kg BaP in rats. Symbols represent (**a**) the average blood concentrations reported in the in vivo studies of Moreau and Bouchard (2015) for intravenous (filled diamonds) and Marie et al. (2010) (open circles) for intravenous exposure. Symbols represent (**b**) the average blood concentrations reported in the in vivo studies of Moreau and Bouchard (2015) (grey open diamonds) and scaled Moreau and Bouchard data based on the fold difference observed in intravenous data (grey open triangles) upon intratracheal exposure. Symbols represent (**c**) the average blood concentrations reported in in vivo studies of Moreau and Bouchard (2015) (black open diamonds) and scaled Moreau and Bouchard data based on the fold difference observed in intravenous data (black open triangles) upon oral exposure. The lines represent the model predicted blood concentrations upon (**a**) intravenous (black solid line), (**b**) intratracheal (grey solid line) and (**c**) oral (black dashed line) exposure

Given that 3-OHBaP induces developmental toxicity in the EST (Kamelia et al. 2020) and that the aim of the present study was to translate in vitro EST data on 3-OHBaP to an in vivo dose–response curve for developmental toxicity of BaP, evaluation of the BaP PBK model focussed on the accuracy of predicting the 3-OHBaP levels. Figure 5 presents the model predictions for BaP and their comparison to the literature reported values for blood BaP concentrations upon (a) intravenous, (b) intratracheal and (c) oral exposure to a dose of 10 mg BaP/kg bw/day. Figure 6 shows a comparison of the predicted time-dependent blood concentrations of 3-OHBaP to reported in vivo time-dependent blood concentrations of 3-OHBaP in rats upon (a) intravenous, (b) intratracheal and (c) oral exposure to a dose of 10 mg BaP/kg bw/day. The data presented in Figs. 5a and  6a reveal that the model predictions for both BaP and 3-OHBaP match the experimental data of Marie et al. (2010) well, while the data of Moreau and Bouchard (2015), which were obtained at a similar dose level, report blood concentrations that are substantially lower than those reported by Marie et al. and/or the predictions. Table 6 summarises the maximum blood concentrations (*C*_max_) of 3-OHBaP in rat blood reported in vivo and predicted by the PBK model. Although in both experimental in vivo studies rats of the same strain were exposed intravenously to the same dose of 10 mg/kg bw/day, the predicted *C*_max_ values differed. As a result, the *C*_max_ reported by Marie et al. (2010) was 1.1 fold higher and that of Moreau and Bouchard (2015) was 3.1 fold lower than the predicted *C*_max_. The predicted *C*_max_ of 3-OHBaP was 4.6 and 9.6 fold higher upon intratracheal and oral exposure to BaP, respectively, compared to the *C*_max_ of 3-OHBaP reported in the study of Moreau and Bouchard (2015) (Fig. 6b, c). The study by Marie et al. (2010) did not report data for intratracheal or oral dosing, and as a result, a comparison between predictions and experimental data for these routes of administration could only be made using the data of Moreau and Bouchard. When correcting these experimental data for intratracheal and oral dosing using the factor difference observed between the two experimental data sets upon intravenous dosing, the scaled experimental data of Moreau and Bouchard match the model predictions much better. The predicted *C*_max_ of 3-OHBaP was 0.8 and 1.9 fold higher upon intratracheal and oral exposure to BaP, respectively, compared to the scaled *C*_max_ of 3-OHBaP reported in the study of Moreau and Bouchard (2015). The apparent discrepancy between the two available in vivo studies is not surprising considering that the mean value of 3-OHBaP recovery in the Moreau and Bouchard study was reported to be relatively low (43% in blood).

**Table 6:** Maximum blood concentration (*C*_max_) of 3-OHBaP in rat blood reported in vivo and predicted by the PBK model upon intravenous, intratracheal and oral administration of 10 mg/kg bw BaP

References	Route of exposure	*C*_max_ predicted (µM)	*C*_max_ reported (µM)	*C*_max_ predicted/*C*_max_ reported
Moreau and Bouchard (2015)	Intravenous	0.68	0.22	3.1
Marie et al. (2010)	Intravenous	0.68	0.72	0.9
Moreau and Bouchard (2015)	Intratracheal	0.65	0.14	4.6
Scaled Moreau and Bouchard (2015)	Intratracheal	0.65	0.77	0.8
Moreau and Bouchard (2015)	Oral	0.96	0.10	9.6
Scaled Moreau and Bouchard (2015)	Oral	0.96	0.51	1.9

Based on these observations, it was concluded that the PBK model predicted the plasma *C*_max_, for 3-OHBaP well enough for further use and evaluation of the PBK model using it for PBK model based reverse dosimetry. The predictions made by the PBK model based reverse dosimetry may then also be used to further evaluate the model and its predictions.

To enable prediction of repeated dose exposures, the PBK model was extended to allow repeated daily dosing. Steady state in the blood concentration of 3-OHBaP upon repeated exposure to BaP was reached after approximately 15 repetitions for all 3 routes of exposure. The *C*_max_ values for 3-OHBaP predicted for repeated intravenous, intratracheal and oral exposure to BaP were 1.7, 1.7 and 1.4 times higher than the predicted *C*_max_ values upon a single BaP dose for these three routes of exposure. A figure presenting the model predicted time-dependent blood concentrations for repeated intravenous, intratracheal and oral exposure to BaP is included in supplementary materials 2 (figure S1).

**Fig. 7:**
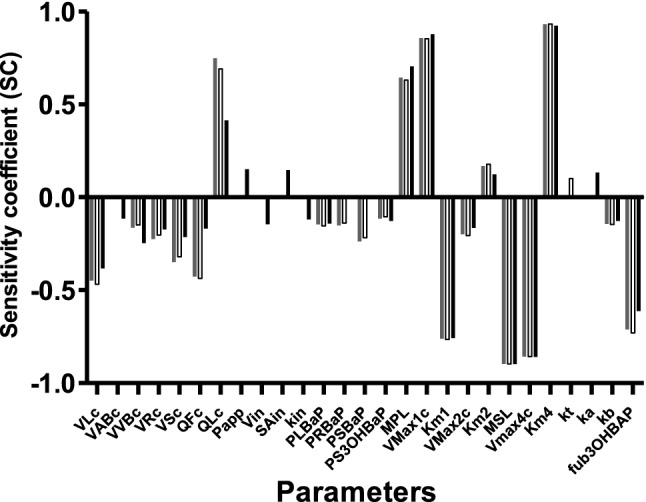
Sensitivity coefficients (SC) of PBK model parameters for the predicted Cmax of 3-OHBaP in rat blood after intravenous (grey bars), oral (black bars) or intratracheal (white bars) administration of 10 mg/kg bw BaP. Model parameters with an absolute SC of ≥ 0.1 are shown. VLc = fraction of liver tissue, VABc = fraction of arterial blood, VVBc = fraction of venous blood, VRc = fraction of rapidly perfused tissue, VSc = fraction of slowly perfused tissue, QFc = fraction of blood flow to fat, QLc = fraction of blood flow to liver, QLUc = fraction of blood flow to lung, Papp = apparent intestinal permeability coefficient in vitro obtained in the Caco-2 model, Vin = volume of each compartment of intestines, SAin = surface area intestinal compartment, kin = transfer rate to next compartment within the intestines, PLBaP = liver/blood partition coefficient of BaP, PRBaP = rapidly perfused tissue:blood partition coefficient of BaP, PSBaP = slowly perfused tissue: blood partition coefficient of BaP, PS3OHBaP = slowly perfused tissue:blood partition coefficient of 3-OHBaP, MPL = microsomal protein content in liver, Vmax1c = maximum rate of 3-OHBaP formation in liver, Km1 = Michaelis–Menten constant for metabolism of BaP to 3-OHBaP in liver, Vmax2c = maximum rate formation of other metabolites in liver, Km2 = Michaelis–Menten constant for metabolism of BaP to other metabolites, MSL = S9 protein content in liver, Vmax4c = maximum rate of glucuronidation of 3-OHBaP formation in liver, Km4 = Michaelis–Menten constant for glucoronidation 3-OHBaP in liver, kt = absorption constant from tracheal to lung of BaP, ka = absorption constant stomach of BaP to GI-tract, kb = excretion constant bile to faeces of BaP, fubBaP = fraction unbound of BaP, fub3OHBaP = fraction unbound of 3-OHBaP

A sensitivity analysis was performed to identify the parameter values that have the highest influence on the model simulations for the *C*_max_ of 3-OHBaP in blood upon intratracheal, intravenous and oral exposure to 10 mg/kg bw BaP. Sensitivity coefficients with an absolute value of 0.1 and higher are shown in Fig. 7. Parameters related to fractional blood flow to liver tissue (QLc), the fraction unbound of 3-OHBaP (fub3OHBaP), microsomal and S9 protein content of the liver (MPL and MSL) and kinetic parameters for the metabolism of BaP and glucuronidation of 3-OHBaP (*K*_m1_, *K*_m4_,* V*_max1c,_ *V*_max4c_) were found to be most influential on the simulated *C*_max_ of 3-OHBaP.

### Translating in vitro concentration–response data into in vivo dose–response data using PBK modelling-based reverse dosimetry

**Fig. 8:**
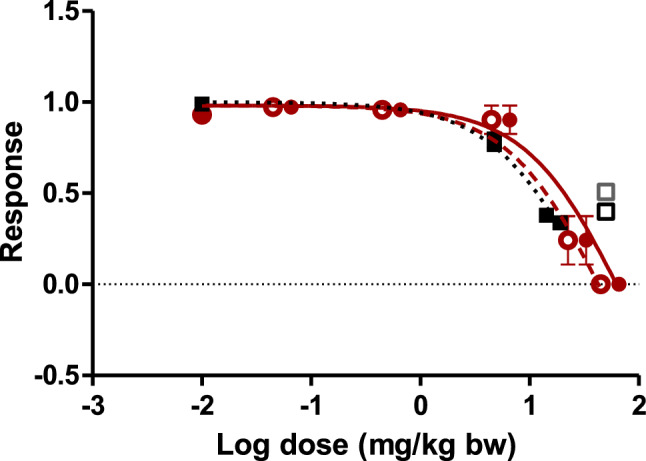
Model predicted and reported in vivo dose–response curves for developmental toxicity of BaP in rats upon single and repeated oral exposure. The predicted dose–response curves are translated from the concentration response curve obtained in the EST for 3-OHBaP (Kamelia et al. 2020) representing single oral exposure (filled red circles and red solid line) and repeated oral exposure (red open circles and red dashed line). Filled black squares and dotted black line represent the in vivo data of Archibong et al. (2002), the open squares represent the data of Bui et al. (1986) for 3 day exposure (black open square) and 6 day exposure (grey open square). The error bars represent the standard error of the mean (SEM) (color figure online)

Using the PBK model thus obtained and evaluated, the in vitro concentration–response data from the EST for 3-OHBaP were translated into a dose–response curve for the developmental toxicity of BaP, after correction for differences in free fraction of 3-OHBaP in vivo and in vitro. Differences in protein binding between the in vitro and in vivo situation where corrected for as described in the materials and methods in "Translating in vitro concentration–response data into in vivo dose–response data using PBK modelling-based reverse dosimetry" section with the values for *f*_ub, in vitro_ of 0.007 and *f*_ub, in vivo_ of 0.0035. This correction provides the total blood *C*_max_ value that matches the total in vitro concentration in the EST corrected for protein binding. Using a curve relating, the PBK model predicted total blood *C*_max_ values of 3-OHBaP to the oral dose levels of BaP, the EST concentration–response curve for in vitro developmental toxicity of 3-OHBaP was converted to an in vivo dose–response curve for developmental toxicity of BaP. The predicted dose–response curve for single exposure to BaP thus obtained is shown in Fig. 8. In addition, Fig. 8 also presents the dose–response curve predicted based on the steady-state *C*_max_ values obtained upon repeated oral exposure to BaP.

## Evaluation of the PBK modelling-based reverse dosimetry based predictions

To evaluate the predicted dose–response curves for developmental toxicity of BaP, Fig. 8 also presents the in vivo dose–response data on reproductive toxicity of BaP, represented by the foetal survival, reported in the literature upon exposure of rats to BaP via nasal inhalation for 10 days (Archibong et al. 2002) and for subcutaneous BaP exposure for 3 and 6 days in rats (Bui et al. 1986). For this comparison, the inhalation dose levels in the study of Archibong et al. (2002) were expressed in equivalent oral dose levels in mg/kg bw per day reported previously (Hood et al. 2000; Ramesh et al. 2002) (Table 3). Visual comparison of the dose–response curves reveals that the predicted dose–response curves are in agreement with the experimental data. This further evaluates the PBK model for prediction of 3-OHBaP *C*_max_ values upon exposure to BaP.

The ED_50_ values derived from the predicted and reported dose–response curves, amount to 67 mg/kg bw for single oral exposure to BaP, 45 mg/kg bw per day for repeated oral exposure to BaP and 29 mg/kg bw per day for the reported in vivo data (Archibong et al. 2002). From the in vivo data of Bui et al. (1986), no ED_50_ values could be calculated, but the reported effect doses are in line with the model predictions, corroborating the accuracy of the predicted dose–response curves.

## Discussion

In vivo studies have demonstrated the reproductive toxicity of BaP (Archibong et al. 2002, 2012; Bui et al. 1986). The aim of the present study was to evaluate the use of an in vitro–in silico approach using PBK model-facilitated reverse dosimetry for predicting the developmental toxicity of BaP based on in vitro toxicity data from the EST for 3-OHBaP, the main metabolite of BaP responsible for the developmental toxicity of BaP in the EST (Kamelia et al. 2020). The intermediate role of 3-OHBaP in the developmental toxicity of BaP follows from the fact that BaP itself tested negative in the EST without bioactivation (Kamelia et al. 2020). Previous studies have shown that in vivo developmental toxicity can adequately be predicted using reverse dosimetry based on the EST for several compounds, including both compounds for which the developmental toxicity is ascribed to the parent compound itself as well as compounds for which the effect is due to a metabolite (Li et al. 2017a; Louisse et al. 2015; Strikwold et al. 2013, 2017). So far, however, this approach has not been assessed for PAHs, although PAH-containing petroleum substances make up a large part of chemicals that require developmental toxicity testing under REACH. The current study shows that the in vitro–in silico approach is suitable for predicting developmental toxicity of BaP in rats, based on in vitro data of the EST for its major metabolite 3-OHBaP, responsible for the developmental toxicity in vitro.

For reverse dosimetry, EST data for 3-OHBaP and not BaP were used, as the ES-D3 cells of the EST appear to lack sufficient bioactivation activity to convert BaP into the active metabolite 3-OHBaP, explaining why BaP tested negative in the EST (Kamelia et al. 2020). Previous studies confirmed this explanation, since only pre-incubation of BaP with hamster liver microsomes prior to testing in the EST, resulted in a positive response that reflected the level of 3-OHBaP formation in the pre-incubation (Kamelia et al. 2020). These findings explicate the need for including bioactivation of BaP to its reactive metabolite 3-OHBaP, and further metabolism and clearance of 3-OHBaP in the quantitative in vitro–in vivo extrapolation (QIVIVE) for evaluation of the developmental toxicity of BaP.

The metabolism of BaP and 3-OHBaP was represented in the current PBK model by the *V*_max_ and *K*_m_ determined in in vitro incubations with subcellular fractions. Similar studies using rat lung microsomes revealed that pulmonary conversion of BaP into 3-OHBaP was negligible compared to the conversion in the liver. This finding was supported by previous results from microsomal incubations, showing that the metabolic rate of metabolite formation from BaP in rat lung was only 0.008% of liver metabolism (Prough et al. 1979). Further results obtained in the present study for conjugation of 3-OHBaP in incubations with rat liver and lung S9 revealed that also the conjugation of 3-OHBaP mainly occurs in liver. Thus, in the PBK model, formation and clearance of 3-OHBaP was modelled in the liver compartment, while conversion in the lung was considered negligible and not included in the model code.

The PBK model developed was evaluated based on available in vivo data for 3-OHBaP blood concentrations as measured upon dosing 10 mg BaP/kg bw by various routes of administration, including intravenous, intratracheal and oral exposure (Marie et al. 2010; Moreau and Bouchard 2015). BaP and 3-OHBaP are highly lipophilic compounds, as reflected by their high log*P* value. Evaluation of the BaP PBK model was focused on adequate prediction of 3-OHBaP levels, as 3-OHBaP is the main inducer of developmental toxicity in vitro and the objective of the present study was to translate in vivo EST data for 3-OHBaP into an in vivo dose–response curve for BaP (Kamelia et al. 2020). Comparison of the model predictions to the available in vivo data revealed that the model somewhat overpredicted the blood concentrations of 3-OHBaP reported by Moreau and Bouchard (2015), while it adequately predicted the data reported by Marie et al. (2010) and the scaled Moreau and Bouchard data. Given that both in vivo studies were performed in the same strain of rat, using the same route of administration and a similar BaP dose level, it appears that the deviations between the model predictions and the reported in vivo data may originate to a substantial extent from variants between the two experimental data sets.

The results from microsomal incubation of BaP reveal that the *V*_max_ and catalytic efficiency for formation of 3-OHBaP are 5.7- to 54-fold and 3.3- to 78- folds higher than for formation of the other metabolites. These results indicate that the other metabolic routes play a less prominent role than 3-OHBaP formation in the metabolism of BaP. Furthermore, comparison of the in vitro developmental toxicity in the EST of 3-OHBaP and a mixture of BaP metabolites formed in an incubation of BaP with hamster liver microsomes revealed that the in vitro developmental toxicity of the mixture of BaP metabolites could be fully ascribed to the level of 3-OHBaP in this metabolite mixture (Kamelia et al. 2020). Given these results and considerations, the model was used for translation of the in vitro EST data for 3-OHBaP into a predicted in vivo dose–response curve for developmental toxicity of BaP, using PBK model-facilitated reverse dosimetry. The ED_50_ values derived from the predicted in vivo dose–response curve for single and repeated oral exposure thus obtained, were in agreement with the ED_50_ value calculated for the in vivo data of Archibong et al. (2002) and in line with the in vivo data of Bui et al. (1986). These results further support the validity of the PBK model for predicting in vivo 3-OHBaP blood concentrations. The results also indicate that the in vitro–in silico approach provides an adequate estimate of the developmental toxicity of BaP in rats. In spite of this, it is relevant to note that the EST detects development of embryonic stem cells into beating cardiomyocytes and may not reflect the specific sensitive endpoints of developmental toxicity observed upon BaP exposure in animal studies, such as developmental neurotoxicity (Li et al. 2012; McCallister et al. 2008; Sheng et al. 2010; Wormley et al. 2004). Inclusion of in vitro assays representing additional developmental toxicity endpoints may enhance the currently developed approach, broadening its applicability for toxicological risk assessment to an even further extent. It should be acknowledged that the endpoint characterised, the exposure regimen used and the window of sensitivity of the EST protocol may not match the exposure scenario, endpoint characterised and window of sensitivity for a compound when detecting its developmental toxicity in an in vivo study. These discrepancies may be a factor contributing to differences between predicted dose–response curves and observed dose–response data. Furthermore, when translating EST data to in vivo dose–response curves and comparing the predictions thus obtained to in vivo data on developmental toxicity, it is worth to note that in vivo studies may report a variety of endpoints including for example cardiac malformations, resorptions, fetal body weight decrease, skeletal malformation, visceral malformations and also fetal deaths. In previous studies we have shown that the EST in vitro data can be used to adequately predict these different in vivo developmental toxicity endpoints (Kamelia et al. 2017; Li et al. 2016, 2017b; Louisse et al. 2010, 2011, 2015; Strikwold et al. 2012, 2017).

In conclusion, the present study shows that the developed PBK modelling-based reverse dosimetry approach can adequately predict in vivo developmental toxicity of BaP based on in vitro data from the EST for 3-OHBaP, the metabolite responsible for this adverse effect. The predicted ED_50_ values adequately reflected the ED_50_ value calculated from the in vivo data. This study provides a proof of principle for an integrated in vitro–in silico approach for predicting in vivo developmental toxicity of BaP. The method may provide a promising strategy for predicting the developmental toxicity of related polycyclic aromatic hydrocarbons (PAHs), without the need for animal testing.

## Supplementary Information

Below is the link to the electronic supplementary material.

Supplementary file1 (DOCX 27 KB)

Supplementary file2 (DOCX 97 KB)

## Declarations

### Availability of data and materials

Supplementary materials (model code and supplementary figures).

### Code availability

Model code available in supplementary materials.
