# Supplementary material for: Predicting the in vivo developmental toxicity of benzo[a]pyrene (BaP) in rats by an in vitro–in silico approach
Source: Arch Toxicol. 2021 Aug 25;95(10):3323–40. doi: 10.1007/s00204-021-03128-7 (PMC8448719; doi:10.1007/s00204-021-03128-7)
Supplement: Supplementary file 1 — Supplementary file1 (DOCX 27 KB) [file 204_2021_3128_MOESM1_ESM.docx]

;Date:22-01-2021

;Purpose: PBK model benzo[a]pyrene and 3-hydroxybenzo[a]pyrene, built with literature, in vitro and in silico derived parameter values

;Species: Rat (gender mixed)

;Compiled by: Maartje Rietdijk and Danlei Wang, based on PBK models for BaP that included submodels for 3OHBaP reported in the literature (Crowell et al. 2011, Heredia-Ortiz and Bouchard 2013, Campbell et al. 2016)

;==========================================================================

;Physiological parameters

;==========================================================================

;tissue volumes (Crowell et al. (2011), based on Brown et al. (1997))

BW = 0.245 {Kg} ; body weight rat (variable, dependent on study)

VFc = 0.065 ; fraction of fat tissue

VLc = 0.037 ; fraction of liver tissue

VLuc= 0.005 ; fraction of lung tissue

VABc =0.0257 ; fraction of arterial blood

VVBc = 0.0514 ; fraction of venous blood

VRc = 0.2159 ; fraction of richly perfused tissue (= 1 - (VFc + VLc + VLuc + VABc + VVBc+ VSc))

VSc = 0.6 ; fraction of slowly perfused tissue

VF = VFc*BW {L or Kg} ; volume of fat tissue (calculated)

VL = VLc*BW {L or Kg} ; volume of liver tissue (calculated)

VLU = VLuc*BW {L or Kg} ; volume of lung tissue (calculated)

VAB = VABc*BW {L or Kg} ; volume of arterial blood (calculated)

VVB = VVBc*BW {L or Kg} ; volume of venous blood (calculated)

VR = VRc*BW {L or Kg} ; volume of richly perfused tissue (calculated)

VS = VSc*BW {L or Kg} ; volume of slowly perfused tissue (calculated)

;--------------------------------------------------------------------------------------------------------------------

;blood flow rates (Crowell et al. (2010), based on Brown et al. (1997))

QC = 15*BW^0.74 {L/hr} ; cardiac output

QFc = 0.07 ; fraction of blood flow to fat

QLc = 0.183 ; fraction of blood flow to liver

QLuc = 1 ; fraction of blood flow to lung

QRc = 0.4 ; fraction of blood flow to richly perfused tissue

QSc = 0.347 ; fraction of blood flow to slowly perfused tissue

QF = QFc*QC {L/hr} ; blood flow to fat tissue (calculated)

QL = QLc*QC {L/hr} ; blood flow to liver tissue (calculated)

QLu = QLuc*QC {L/hr} ; blood flow to lung tissue (calculated)

QS = QSc*QC {L/hr} ; blood flow to slowly perfused tissue (calculated)

QR = QRc*QC {L/hr} ; blood flow to richly perfused tissue (calculated)

;--------------------------------------------------------------------------------------------------------------------

;Intestinal lumen volumes, surfaces, absorption rates, transfer rates

;Papp, Caco-2 = 3.8 {x1E-6 cm/sec} ;in vitro, from Caco-2 cells (Goth-Goldstein et al. (1999)

PappCaco2=-5.42 ; Log Papp, Caco-2

;Log (Papp,in vivo) = 0.6836*Log(PappCaco-2)-0.5579 (Sun et al. 2002)

Papp=10^(0.6836*PappCaco2-0.5579)*3600/10 ; apparent intestinal permeability coefficient in vivo {dm/hr}

; 7-compartment model for GI-tract based on model bisphenol A and 17β-estradiol by Zhang et al., (2018)

Vin = 0.0012 ; volume for each compartment of intestines {L}

SAin = 0.134 ; surface area {dm2}

kin = 4.17 ; transfer rate to next compartment within the intestines {/hr}

;kabin1 = Papp*SAin ; absorption rate constant {L/hr}

Vin1 = Vin ; volume of intestine compartment 1 {L}

SAin1 = SAin ; surface area of intestine compartment 1 {dm2}

kabin1 = Papp*SAin1 ; absorption rate constant of intestine compartment 1 {L/hr}

kin1 = kin ; transfer rate to intestine compartment 2 {/hr}

Vin2 = Vin ; volume of intestine compartment 2 {L}

SAin2 = SAin ; surface area of intestine compartment 2 {dm2}

kabin2 = Papp*SAin2 ; absorption rate constant of intestine compartment 2 {L/hr}

kin2 = kin ; transfer rate to intestine compartment 3 {/hr}

Vin3 = Vin ; volume of intestine compartment 3 {L}

SAin3= SAin ; surface area of intestine compartment 3 {dm2}

kabin3 = Papp*SAin3 ; absorption rate constant of intestine compartment 3 {L/hr}

kin3 = kin ; transfer rate to intestine compartment 4 {/hr}

Vin4 = Vin ; volume of intestine compartment 4 {L}

SAin4 = SAin ; surface area of intestine compartment 4 {dm2}

kabin4 = Papp*SAin4 ; absorption rate constant of intestine compartment 4 {L/hr}

kin4 = kin ; transfer rate to intestine compartment 5 {/hr}

Vin5 = Vin ; volume of intestine compartment 5 {L}

SAin5 = SAin ; surface area of intestine compartment 5 {dm2}

kabin5 = Papp*SAin5 ; absorption rate constant of intestine compartment 5 {L/hr}

kin5 = kin ; transfer rate to intestine compartment 6 {/hr}

Vin6 = Vin ; volume of intestine compartment 6 {L}

SAin6 = SAin ; surface area of intestine compartment 6 {dm2}

kabin6 = Papp*SAin6 ; absorption rate constant of intestine compartment 6 {L/hr}

kin6 = kin ; transfer rate to intestine compartment 7 {hr}

Vin7 = Vin ; volume of intestine compartment 7 {L}

SAin7 = SAin ; surface area of intestine compartment 7 {dm2}

kabin7 = Papp*SAin7 ; absorption rate constant of intestine compartment 7 {L/hr}

kin7 = kin ; transfer rate to co {/hr}

kfe = 0.27 ; transfer rate to faeces {/hr} taken from Crowell et al. (2011)

;=====================================================================

;Physicochemical parameters

;=====================================================================

;partition coefficients BaP (tissue:blood) (Crowell et al., (2011) calculated according to Poulin and Theil, (2002))

PFBaP = 496.38 ; fat/blood partition coefficient

PLBaP = 13.31 ; liver/blood partition coefficient

PLuBaP = 13.31 ; lung/blood partition coefficient

PRBaP= 13.31 ; rapidly perfused tissue/blood partition coefficient

PSBaP = 6.99 ; slowly perfused tissue/blood partition coefficient

;--------------------------------------------------------------------------------------------------------------------

;partition coefficients 3-OHBaP (tissue:blood) (Crowell et et al. (2011), calculated according to Poulin & Krishnan (1995))

PF3OHBaP = 401 ; fat/blood partition coefficient of 3-OHBaP

PL3OHBaP = 12.24 ; liver/blood partition coefficient of 3-OHBaP

PLu3ohBaP = 12.24 ; lung/blood partition coefficient

PR3OHBaP = 12.24 ; rapidly perfused tissue/blood partition coefficient of 3-OHBaP

PS3OHBaP = 6.43 ; slowly perfused tissue/blood partition coefficient of 3-OHBaP

;=====================================================================

;Kinetic parameters

;=====================================================================

;Metabolism liver

;MPL: scaling factor of rat liver microsomes (mg microsomal protein /g liver)

MPL=45 ; mg microsomal protein/g liver Reference: (Houston & Galetin (2008))

;Maximum rate of metabolism of BaP to 3-OHBaP, measured in vitro in present study

VMax1c= 0.16 {nmol/min/mg microsomal protein} ; BaP -> 3-OHBaP

;Maximum rate of metabolism scaled to liver

VMax1 = VMax1c/1000*60*MPL*VL*1000 {µmol/hr/liver}

;Michaelis-Menten constant for metabolism of BaP to 3-OHBaP measured in vitro in present study

Km1 = 34 {uM}

;--------------------------------------------------------------------------------------------------------------------

;Maximum rate of metabolism of BaP to remaining metabolites measured in present study

VMax2c = 0.13 {nmol/min/mg microsomal protein} ; (BaP-->remaining metabolites)

;Maximum rate of metabolism scaled to liver

VMax2 = VMax2c/1000*60*MPL*VL*1000 {µmol/hr/liver}

;Michaelis-Menten constant for metabolism of BaP to remaining metabolites measured in vitro in present study

Km2 = 17 {uM}

;--------------------------------------------------------------------------------------------------------------------

;Sulfonation of 3-OHBaP

;MSL: scaling factor of rat liver S9 (mg S9 protein /g liver)

MSL = 125 ; mg S9 protein/g liver (Houston and Galetin, 2008)

;Maximum rate of metabolism for sulfation of 3-OHBaP determined in vitro in present study

VMax3c= 0.48 {nmol/min/mg S9 protein}

VMax3 = VMax3c/1000*60*MSL*VL*1000 {µmol/hr/liver}

;Michaelis-Menten constant for sulfation of 3-OHBaP determined in vitro in present study

Km3 = 17 {µM}

;--------------------------------------------------------------------------------------------------------------------

;Glucuronidation of 3-OHBaP

;Maximum rate of metabolism glucuronidation of 3-OHBaP determined in vitro in present study

Vmax4c= 5.7 {nmol/min/mg S9 protein} ;

Vmax4 = Vmax4c/1000*60*MSL*VL*1000 {µmol/hr/liver}

; Michaelis-Menten constant for glucuronidation of 3-OHBaP determined in vitro

Km4 = 10 {µM}

;=====================================================================

;Run settings

;=====================================================================

;Exposure parameters

;Molecular weight

MWBaP = 252.31 ; Molecular weight BaP

MW3OHBaP = 268.3 ; Molecular weight 3-OHBaP

;IV dose = given IV dose in mg/kg bw

IVDOSEmg = 0 {mg/kg bw}

IVDOSEumol2 = IVDOSEmg*1E-3/MWBaP*1E6 {µmol/ kg bw}

;IVDOSEumol2 = given iv dose recalculated to µmol/kg bw

IVDOSEumol=IVDOSEumol2*BW {µmol} ; intravenous dose

;oral dose = given oral dose in mg/kg

ODOSEmg = 0 {mg/kg bw}

ODOSEumol2 = ODOSEmg*1E-3/ MWBAP*1E6 {µmol/kg bw}

ODOSEumol=ODOSEumol2*BW; {µmol} ; oral dose

;intratracheal dose = given intratracheal dose in mg/kg

ITDOSEmg = 0 {mg/kg bw}

ITDOSEumol2 = ITDOSEmg*1E-3/MWBaP*1E6 {µmol/ kg bw}

;ITDOSEumol = given intratracheal dose recalculated to µmol/kg bw

ITDOSEumol=ITDOSEumol2*BW {µmol} ; intratracheal dose

;Time

Starttime = 0 ; in hr

Stoptime = 24 ; in hr

;=====================================================================

;Model calculations

;=====================================================================

;Exposure routes

Frequency = 24 {h} ; duration between doses, in hours

Repetitions = 1 ; total number of daily doses

;Intravenous, needle

;ANe = amount BaP in needle

ANe' = -kd*ANe + iv_input'

Init ANe = 0

Iv_input' = IF time <= Repetitions * Frequency THEN pulse(IVDOSEumol, 0, Frequency) ELSE 0

Init iv_input = 0

kd=1000000 {/h} ;kd, the transport rate from needle to blood

; intratracheal

;ATr = amount BaP in trachea

ATr' = -kt*ATr + it_input'

Init ATr = 0

It_input' = IF time <= Repetitions * Frequency THEN pulse(ITDOSEumol, 0, Frequency) ELSE 0

Init it_input = 0

kt = 1 {h} ; absorption from trachea to lung, maximum value assumed

;--------------------------------------------------------------------------------------------------------------------

;Stomach compartment

;Ast = amount of BaP remaining in stomach, µmol

Ast' = -ka*Ast + oral_input'

Init Ast = 0

oral_input' = IF time <= Repetitions * Frequency THEN pulse(ODOSEumol, 0, Frequency) ELSE 0

Init oral_input = 0

ka = 1 {h} ; Absorption constant stomach to GI-tract maximum value assumed

;--------------------------------------------------------------------------------------------------------------------

;intestinal compartment, divided in 7 sub compartments

;Ain1 = Amount BaP in intestine compartment 1 (µmol)

Cin1 = Ain1/Vin1

Ain1' = ka*Ast - kin1*Ain1 - kabin1*Cin1

Init Ain1 = 0

;Ain2 = Amount BaP in intestine compartment 2 (µmol)

Cin2 = Ain2/Vin2

Ain2' = kin1*Ain1 - kin2*Ain2 - kabin2*Cin2

Init Ain2 = 0

;Ain3 = Amount BaP in intestine compartment 3 (µmol)

Cin3 = Ain3/Vin3

Ain3' = kin2*Ain2 - kin3*Ain3 - kabin3*Cin3

Init Ain3 = 0

;Ain4 = Amount BaP in intestine compartment 4 (µmol)

Cin4 = Ain4/Vin4

Ain4' = kin3*Ain3 - kin4*Ain4 - kabin4*Cin4

Init Ain4 = 0

;Ain5 = Amount BaP in intestine compartment 5 (µmol)

Cin5 = Ain5/Vin5

Ain5' = kin4*Ain4 - kin5*Ain5 - kabin5*Cin5

Init Ain5 = 0

;Ain6= Amount BaP in intestine compartment 6 (µmol)

Cin6 = Ain6/Vin6

Ain6' = kin5*Ain5 - kin6*Ain6 - kabin6*Cin6

Init Ain6 = 0

;Ain7= Amount BaP in intestine compartment 7 (µmol)

Cin7 = Ain7/Vin7

Ain7' = kin6*Ain6 - kin7*Ain7 - kabin7*Cin7

Init Ain7 = 0

;Aco = Amount BaP in colon (µmol)

Aco' = kin7*Ain7- kfe*Aco

Init Aco = 0

ACco' = kin7*Ain7

Init ACco = 0 ; cumulative amount reaching colon

;--------------------------------------------------------------------------------------------------------------------

;feces

;AFA = amount BaP in feces (µmol)

AFe' = kfe*Aco + Kb*ALBAP

Init AFe = 0

Kb = 1 ; excretion constant liver to faeces via bile maximum value assumed

;--------------------------------------------------------------------------------------------------------------------

;liver compartment

;ALBAP = Amount of BaP in liver tissue (µmol)

ALBAP' = kabin1*Cin1 + kabin2*Cin2 + kabin3*Cin3 + kabin4*Cin4 + kabin5*Cin5 + kabin6*Cin6 + kabin7*Cin7 + QL*( CABBAP - CVLBAP) - AMMO'- AMMT1' - Kb*ALBAP

Init ALBAP = 0

CLBAP = ALBAP/VL

CVLBAP = CLBAP/PLBAP

;AMMO = amount of BaP metabolized to metabolite 3-OHBenzo[a]pyrene

AMMO' =VMax1*CVLBAP/(Km1 + CVLBAP)

init AMMO = 0

;AMMT1 = amount of BaP metabolized to other metabolites

AMMT1' = Vmax2*CVLBAP/(Km2 + CVLBAP)

init AMMT1 = 0

;--------------------------------------------------------------------------------------------------------------------

;fat compartment

;AFBAP = Amount of BaP in fat tissue (µmol)

AFBAP' = QF*( CABBAP -CVFBAP)

Init AFBAP = 0

CFBAP = AFBAP/VF

CVFBAP = CFBAP/PFBAP

;--------------------------------------------------------------------------------------------------------------------

;tissue compartment richly perfused tissue

;ARBAP = Amount of BaP in richly perfused tissue (µmol)

ARBAP' = QR*( CABBAP -CVRBAP)

Init ARBAP = 0

CRBAP = ARBAP/VR

CVRBAP = CRBAP/PRBAP

;--------------------------------------------------------------------------------------------------------------------

;tissue compartment slowly perfused tissue

;ASBAP = Amount of BaP in slowly perfused tissue (µmol)

ASBAP' = QS*( CABBAP -CVSBAP)

Init ASBAP = 0

CSBAP = ASBAP/VS

CVSBAP = CSBAP/PSBAP

;--------------------------------------------------------------------

;lung blood compartment

;ALUBAP = Amount of BaP in lung blood, µmol

ALUBAP' = kt*ATr+ QLU*(CVBBAP - CVLUBAP)

Init ALUBAP = 0

CLUBAP = ALUBAP/VLU

CVLUBAP = CLUBAP/PLUBAP

;--------------------------------------------------------------------

; venous blood

;AVBBAP = amount of BaP in venous blood, µmol

AVBBAP' = Ane*kd + (QF*CVFBaP + QL*CVLBaP + QS*CVSBaP + QR*CVRBaP) - QLU*CVBBAP

Init AVBBAP = 0

CVBBAP = AVBBAP/VVB

AUCVBBAP' = CVBBAP

init AUCVBBAP = 0

;--------------------------------------------------------------------

; arterial blood

;AABBAP = amount of BaP in arterial blood, µmol

AABBAP' = QLU*(CVLUBAP -CABBAP)

Init AABBAP = 0

CABBAP = AABBAP/VAB

AUCABBAP' = CABBAP

init AUCABBAP = 0

CBBaPtot =CVBBAP + CABBAP

;========================================================================

;3-hydroxybenzo[a]pyrene submodel

;========================================================================

;feces

;AFA = amount 3OHBaP in feces (µmol)

A3OHFe' = kfe*Aco + Kc*AL3OHBAP

Init A3OHFe = 0

Kc = 1 ;excretion constant liver to faeces via bile maximum value assumed

;--------------------------------------------------------------------------------------------------------------------

;liver compartment

;AL3OHBAP = Amount of 3-OHBaP in liver tissue (µmol)

AL3OHBAP' = AMMO' + QL*( CAB3OHBAP - CVL3OHBAP) - AMMT2' - AMMT3' - Kc*AL3OHBAP

Init AL3OHBAP = 0

CL3OHBAP = AL3OHBAP/VL

CVL3OHBAP = CL3OHBAP/PL3OHBAP

;AMMT2 = Amount of 3-OHBaP sulfonated

AMMT2' = Vmax3*CVL3OHBAP/(Km3 + CVL3OHBAP)

Init AMMT2 = 0

;AMMT3 = Amount of 3-OHBaP glucuronidated

AMMT3' = Vmax4*CVL3OHBAP/(Km4 + CVL3OHBAP)

Init AMMT3 = 0

;--------------------------------------------------------------------------------------------------------------------

;fat compartment

;AF3OHBAP = Amount of 3-OHBaP in fat tissue (µmol)

AF3OHBAP' = QF*( CAB3OHBAP -CVF3OHBAP)

Init AF3OHBAP = 0

CF3OHBAP = AF3OHBAP/VF

CVF3OHBAP = CF3OHBAP/PF3OHBAP

;--------------------------------------------------------------------------------------------------------------------

;tissue compartment richly perfused tissue

;AR3OHBAP = Amount of 3-OHBaP in richly perfused tissue (µmol)

AR3OHBAP' = QR*( CAB3OHBAP -CVR3OHBAP)

Init AR3OHBAP = 0

CR3OHBAP = AR3OHBAP/VR

CVR3OHBAP = CR3OHBAP/PR3OHBAP

;--------------------------------------------------------------------------------------------------------------------

;tissue compartment slowly perfused tissue

;AS3OHBAP = Amount of 3-OHBaP in slowly perfused tissue (µmol)

AS3OHBAP' = QS*( CAB3OHBAP -CVS3OHBAP)

Init AS3OHBAP = 0

CS3OHBAP = AS3OHBAP/VS

CVS3OHBAP = CS3OHBAP/PS3OHBAP

;--------------------------------------------------------------------

;lung compartment

;ALU3OHBAP = Amount of 3-OHBaP in lung tissue, µmol

ALU3OHBAP' = QLU*(CVB3OHBAP -CVLU3OHBAP)

Init ALU3OHBAP = 0

CLU3OHBAP = ALU3OHBAP/VLU

CVLU3OHBAP = CLU3OHBAP/PLU3OHBAP

;--------------------------------------------------------------------

; venous blood

;AVB3OHBAP = amount of 3-OHBaP in venous blood, µmol

AVB3OHBAP' = ( (QF*CVF3OHBAP + QL*CVL3OHBAP + QS*CVS3OHBAP + QR*CVR3OHBAP) - QLU*CVB3OHBAP)*fub3OHBAP

Init AVB3OHBAP = 0

CVB3OHBAP = AVB3OHBAP/VVB

AUCVB3OHBAP' = CVB3OHBAP

init AUCVB3OHBAP = 0

fub3OHBAP = 0.007 ; 3-OHBaP fraction unbound in blood

;--------------------------------------------------------------------

; venous blood protein

;AVBP3OHBAP = amount of 3-OHBaP in bound to venous blood protein, µmol

AVBP3OHBAP' = ( (QF*CVF3OHBAP + QL*CVL3OHBAP + QS*CVS3OHBAP + QR*CVR3OHBAP) - QLU*CVB3OHBAP)*fb3OHBAP

Init AVBP3OHBAP = 0

CVBP3OHBAP = AVBP3OHBAP/VVB

AUCVBP3OHBAP' = CVBP3OHBAP

init AUCVBP3OHBAP = 0

fb3OHBaP = 1-fub3OHBAP ; 3-OHBaP fraction bound to blood protein

;--------------------------------------------------------------------

; arterial blood

;AAB3OHBAP = amount of 3-OHBaP in arterial blood, µmol

AAB3OHBAP' = (QLU*(CVLU3OHBAP -CAB3OHBAP)) * fub3OHBAP

Init AAB3OHBAP = 0

CAB3OHBAP = AAB3OHBAP/VAB

AUCAB3OHBAP' = CAB3OHBAP

init AUCAB3OHBAP = 0

;--------------------------------------------------------------------

; arterial blood protein

;AABP3OHBAP = amount of 3-OHBaP bound to arterial blood protein, µmol

AABP3OHBAP' = (QLU*(CVLU3OHBAP - CAB3OHBAP))* fb3OHBAP

Init AABP3OHBAP = 0

CABP3OHBAP = AABP3OHBAP/VAB

CB3OHBaPtot = CVB3OHBAP + CVBP3OHBAP + CAB3OHBAP + CABP3OHBAP

;========================================================================

;Mass balance calculations for Benzo[a]pyrene

TotalBAP = oral_input + iv_input + it_input

CalculatedBAP = Ain1 + Ain2 + Ain3 + Ain4 + Ain5 + Ain6 + Ain7 + Aco + AFe + ALBAP + AFBAP +ASBAP + ARBAP + AABBAP + AVBBAP +ALUBAP + AMMO + AMMT1 + ANe + Ast + ATr

ERRORBAP=(TotalBAP-CalculatedBAP)/(TotalBAP+1E-30)*100

MASSBBALBAP=TotalBAP-CalculatedBAP + 1

;========================================================================

;Mass balance calculations for 3-hydroxybenzo[a]pyrene sub-model

Total3OHBAP = AMMO

Calculated3OHBAP = A3OHFe + AL3OHBAP + AF3OHBAP +AS3OHBAP + AR3OHBAP + AAB3OHBAP + AABP3OHBAP+AVB3OHBAP + AVBP3OHBAP + ALU3OHBAP + AMMT2 + AMMT3

ERROR3OHBAP=(Total3OHBAP-Calculated3OHBAP)/(Total3OHBAP+1E-30)*100

MASSBBAL3OHBAP=Total3OHBAP-Calculated3OHBAP + 1

;========================================================================
