# Supplementary material for: Predicting the in vivo developmental toxicity of benzo[a]pyrene (BaP) in rats by an in vitro–in silico approach
Source: Arch Toxicol. 2021 Aug 25;95(10):3323–40. doi: 10.1007/s00204-021-03128-7 (PMC8448719; doi:10.1007/s00204-021-03128-7)
Supplement: Supplementary file 2 — Supplementary file2 (DOCX 97 KB) [file 204_2021_3128_MOESM2_ESM.docx]

**a**

**b**

**c**

Figure S1 Predicted time-dependent blood concentrations of 3-OHBaP in rats upon repeated (a) intravenous, (b) intratracheal and (c) oral exposure to 10 mg/kg/day BaP in rats.
